# INcentives and ReMINDers to Improve Long‐Term Medication Adherence (INMIND): impact of a pilot randomized controlled trial in a large HIV clinic in Uganda

**DOI:** 10.1002/jia2.26306

**Published:** 2024-06-25

**Authors:** Sebastian Linnemayr, Mary Odiit, Barbara Mukasa, Ishita Ghai, Chad Stecher

**Affiliations:** ^1^ RAND Corporation Santa Monica California USA; ^2^ Mildmay Uganda Kampala Uganda; ^3^ College of Health Solutions Arizona State University Phoenix Arizona USA

**Keywords:** medication adherence, HIV, antiretroviral therapy, habit formation, behavioural economics, Uganda

## Abstract

**Introduction:**

Habits are a common strategy for successfully countering medication non‐adherence, yet existing interventions do not support participants during the long habit formation period, resulting in high attrition. We test a novel intervention combining text messages and incentives with anchoring to support antiretroviral therapy (ART) pill‐taking habits.

**Methods:**

In a randomized, parallel controlled trial, a sample of 155 participants 18 years and older who initiated ART within 3 months were recruited at Mildmay Uganda between October 2021 and April 2022. All participants were educated on the anchoring strategy and chose an anchor, that is existing routines, to pair with pill‐taking. Participants were randomized to either usual care (C = 49), daily text message reminders to follow their anchoring plan (*Messages* group; T1 = 49) or messages and incentives conditional on pill‐taking in line with their anchor (*Incentives* group; T2 = 57). Assessments occurred at baseline, month 3 (end of intervention) and month 9 (end of observation period). The primary outcomes are electronically measured mean adherence and pill‐taking consistent with participants’ anchor time.

**Results:**

The primary outcome of pill‐taking in line with the anchoring plan was higher in the *Incentives* group during the 3‐month intervention (12.2 p.p. [95% CI: 2.2 22.2; *p* = .02]), and remained significantly higher after the incentives were withdrawn (months 4−6 (14.2 p.p. [95% CI 1.1 27.2; *p* = .03]); months 7−9 (14.1 p.p. [95% CI −0.2 28.5; *p* = .05])). Mean adherence was higher in both treatment groups relative to the control group during the intervention (T1 vs. C, *p* = .06; T2 vs. C, *p* = .06) but not post‐intervention.

**Conclusions:**

The promising approach of using incentives to support habit formation among ART treatment initiators needs to be evaluated in a fully powered study to further our understanding of the habit formation process and to evaluate its cost‐effectiveness.

## 1 INTRODUCTION

Antiretroviral therapy (ART) has significantly improved disease outcomes and quality of life among people living with HIV [1, 2]. Globally, about 75% of people living with HIV receive ART; however, only 67% of them are virally suppressed [3], and ART medication non‐adherence has emerged as a key adherence barrier even when structural and economic barriers have been addressed [4, 5, 6, 7]. Treatment initiators face specific challenges to achieving healthy long‐term outcomes, such as declining motivation and forgetfulness [8, 9], While some interventions have targeted treatment initiators in low‐ and middle‐income settings, a systematic literature review emphasizes the poor quality of the existing interventions [10].

Habits are a commonly reported strategy for maintaining medication adherence among patients successfully managing chronic diseases like HIV [11, 12]. ART initiation offers a critical intervention window, because it is easier to form a new habit than to break a bad one [13, 14]. Habits are formed over time by performing a new behaviour in response to a contextual cue [13], and a common intervention strategy called “anchoring” is to identify an existing routine (such as brushing one's teeth) as the cue for the new habit [14, 15]. This strategy has improved physical activity, mindfulness meditation and medication adherence [16, 17, 18, 19]. However, existing interventions do not support participants during the 3−4 months it takes to form a new habit, resulting in high attrition, often 40% or more [16, 17, 18, 20, 21].

The behavioural economics (BE) biases of the salience of ART adherence and present bias may be limiting the success of existing anchoring interventions [22, 23, 24]. Salience refers to how prominent a condition is in a person's mind; it tends to be highest at treatment initiation, and decreases over time as other, more pressing needs dominate one's attention. Present bias is the tendency to over‐value immediate rewards at the detriment of long‐term benefits. Two intervention strategies can address these biases: (1) text message reminders can maintain salience; they have been used to support habit formation for dietary choices [25], but not for medication adherence. (2) Incentives for performing health behaviours have improved physical activity [26], smoking cessation [27] and medication adherence [28]. To counter present bias (and subsequent attrition) during medication adherence habit formation, incentives can be made conditional on pill‐taking around the time of the participants’ existing routine, that is providing rewards for cued adherence. This novel incentivization approach has the advantage that the incentives are provided in a targeted, short‐term manner (i.e. only during the 3−4 months of the habit formation process), but with long‐term impact (once the habit is established). It also improves on the key drawback of traditional incentive interventions that typically see behaviours return to baseline levels once incentives are withdrawn [29]. Similarly, text messages provided for the relatively short and clearly defined habit formation period are likely to increase the salience of the targeted behaviour without risk of being perceived as repetitive or annoying.

In this paper, we test the feasibility, acceptability and preliminary efficacy of the pilot intervention “INcentives and ReMINDers to Improve Long‐term Medication Adherence” (INMIND) that uses text message reminders and incentives conditional on cued adherence to establish high ART adherence habits among treatment initiators in a large HIV clinic in Uganda.

## 2 METHODS

### 2.1 Study design and setting

INMIND is a pilot, parallel‐group randomized controlled trial (RCT) with two intervention groups and a control group using an even allocation ratio of 1:1:1. The study was performed at Mildmay Hospital, an HIV clinic in Kampala, Uganda that provides comprehensive HIV and AIDS prevention, care and treatment services to over 25,000 people living with HIV, and has a well‐established electronic medical records (EMRs) infrastructure. The study was approved by RAND's Human's Subjects Protection Committee (2020‐N0632), the Mildmay Uganda Research Ethics Committee (MUREC) (0701‐2021), and the Uganda National Council for Science and Technology (HS128ES). The trial was registered on Clinicaltrials.gov (registration number: NCT05131165) on 12 November 2021. Further details on study procedures can be found in the protocol paper [30].

### 2.2 Participants

The sample consists of male and female clients aged 18 and older who started ART at Mildmay within the preceding 3 months, own or have access to a phone on at least 5 days a week and be willing to receive study text messages. Clients not mentally fit to understand the consenting or study procedures, as well as clients not speaking English or Luganda (the local language spoken by most people in and around Kampala) were excluded. One hundred and sixty‐nine participants were recruited from Mildmay Hospital, roughly representative of the overall patient population with a 70:30 ratio of female to male clients. EMRs were used to screen the client population for initial eligibility based on age and time since ART initiation. Potentially eligible clients interested in study participation had their eligibility verified and gave written consent to participate. Data were collected between October 2021 and April 2022.

### 2.3 Randomization and masking

Assignment to study group was carried out through a computer‐generated randomization component built into the baseline survey administration software called Questionnaire Development System. Participants were randomized 1:1:1 to either a control arm or one of two intervention arms (*Messages*; or *Incentives*). Treatment assignment was revealed at the end of the baseline survey to both the participant and the study coordinator to not influence survey answers. Given the nature of the intervention, neither the interviewers nor the participants could be blinded to the treatment status. However, the data analyst was blinded to the treatment assignment.

### 2.4 Procedures

All participants (including in the control group) received information about the importance of habits, and were then asked to select one of three pill‐taking anchors that were chosen in a preceding formative phase: getting dressed in the morning, having breakfast or eating dinner, together with the time that anchor typically occurs. All participants then created an ART adherence anchoring plan (such as “I will take my medication every day with breakfast around 7 AM”). Adherence was objectively measured using medication event management system (MEMS) caps that electronically record all bottle openings and their timing. All participants (including the control group) received a MEMS cap device to avoid spurious effects brought about by using the device.

In the *Messages* intervention group, participants additionally received daily text message reminders for 3 months to keep adherence and their anchoring strategy salient. Example messages include “Hello, this is INMIND. Remember to stick with your healthy plans!”. Importantly, the texts are not direct pill‐taking reminders that would likely be counter‐productive because when the cue (the text message) is withdrawn, the behaviour (pill‐taking) would no longer be triggered. Accordingly, these text message reminders were sent at 2 PM to avoid any potential overlap with the three anchors: getting dressed in the morning, having breakfast or eating dinner.

In the *Incentives* group, participants received the same messages, but could also become eligible for small incentives conditional on cued pill‐taking, that is for taking their ART pills on 70% or more of the 30 days preceding the study visit within at the time of the selected anchor (such as at 7 AM +/− 1 hour). They could win mobile airtime worth 500, 5000 and 10,000 Ush ($0.15−3 USD) at each of three‐monthly visits during the intervention period. Qualifying participants chose from one of three cards lying face down on a table, and the prize printed on the chosen card was sent to the participant's phone number (via Reloadly) as mobile airtime.

The intervention was administered for 3 months during which MEMS data readings were collected monthly (typically coinciding with the monthly clinic visits mandated for newly diagnosed clients), and prize drawings for eligible participants in the *Incentives* group were carried out. During the 6‐month observation period following the intervention, the team downloaded MEMS data during the participants’ regular clinic visits (typically monthly for the first 6 months post‐treatment initiation, and every 3−6 months thereafter). Surveys were administered at baseline, month 3 (end of intervention period) and month 9 (end of observation period). The intervention was preceded by a formative phase, and succeeded by an adaptation phase to collect qualitative data on the overall design, feasibility and acceptability of the intervention components. See the SPIRIT checklist for a guide to the key items reported in this protocol (Additional File 1).

### 2.5 Measures

#### 2.5.1 Participant characteristics

Participants’ age, number of children, monthly income (USD), and travel cost (USD) and travel time (hours) to the HIV clinic were collected on the baseline survey. Participants’ health limitations were measured using a researcher‐defined question asking if their health kept them working at a job, at home or at school in the past month, with responses on a 6‐point Likert scale from “All of the time” (1) to “None of the time” (6). ART adherence motivation was measured using an adapted version of the Intrinsic Motivation Inventory [31], medication adherence habit strength was measured using the Self‐Reported Behavioral Automaticity Index [32], and participants’ willingness to take risks and willingness to delay rewards were each measured using a single‐item questionnaire developed by Falk et al. [33].

#### 2.5.2 Feasibility and acceptability

The feasibility and acceptability of the INMIND intervention are assessed by the study retention rate and the attendance rate for scheduled clinic visits, as well as through survey measures at months 3 and 9 asking participants about their comfort using the electronic pill bottle caps, the ability to understand all intervention materials and their perceived value of the intervention.

#### 2.5.3 Primary and secondary outcomes

The first primary outcome measure is electronically measured mean medication adherence during the 3‐month intervention period, as well as for 6 months post‐intervention to investigate behavioural persistence. Mean adherence is calculated as the number of pills taken per day, divided by the number of pills prescribed (i.e. # of actual bottle openings / # of prescribed bottle openings), capped at 100%, meaning that any pill bottle openings over the participants’ number of prescribed daily pills were ignored. The second primary outcome is an indicator of cued adherence, calculated as the fraction of pills taken within +/− 1 hour around the time participants report that their chosen anchor occurs. This measure provides an objective way for assessing medication adherence habit formation, and is calculated both during and after the intervention.

We also assessed two secondary outcome measures: retention in care based on Mildmay electronic records, with participants who did not make any clinic visits for 6 months or more considered lost to follow‐up. HIV RNA (viral load) is the other secondary outcome measure, defined as a confirmed viral load of > 200 copies/ml.

### 2.6 Statistical analysis

This pilot study was designed to assess feasibility, acceptability and preliminary efficacy, so the targeted sample size of 150 participants was not chosen to be able to statistically detect the minimum clinically meaningful difference in mean adherence. Instead, we were powered to detect an 11 percentage point difference in mean adherence between study groups (with 80% power at a 5% significance threshold). For our outcome of cued adherence, our sample was powered to detect differences of 11.7 percentage points between study groups with 80% power at a 5% significance threshold.

Feasibility and acceptability were analysed using summary statistics derived directly from the self‐reported measures. To estimate preliminary efficacy, we used an intent‐to‐treat analysis to compare group‐level differences in the primary and secondary outcome measures, performed using multivariate regression models estimated by ordinary least squares (OLS). Regression models for each outcome were first estimated using study group identifiers as the only independent variables, and then a second model was estimated controlling for all observed participant characteristics to improve statistical power. These two models were specified a priori, and the statistical significance of model coefficients was assessed based on heteroskedasticity‐robust standard errors. For analyses of dichotomous outcome measures, such as viral suppression, analogous multivariate logistic regression models were used to assess study group‐level differences. All regression analyses were performed in Stata/MP v16.1, and statistical significance was determined using a threshold of α < .05.

### 2.7 Role of the funding source

The funder of the study had no role in the study design, data collection, data analysis, data interpretation or writing of the report. All authors had full access to all data in the study, and the corresponding author had final responsibility for the decision to submit for publication.

## 3 RESULTS

**Figure 1:**
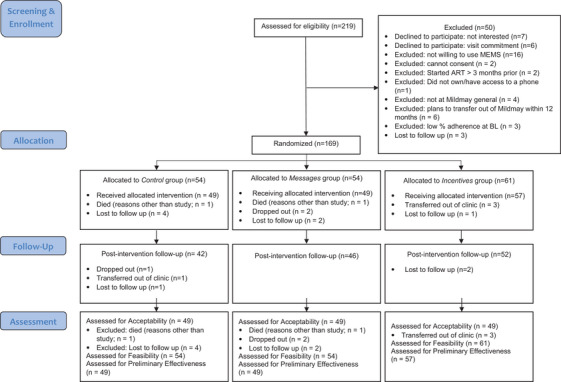
CONSORT flow diagram for INMIND.

A total of 219 Mildmay clients were assessed for study eligibility; 50 were excluded, and the primary reason for non‐participation was unwillingness to use the electronic pill bottle cap. We randomized 169 participants who consented to study participation between the three study groups and analysed the preliminary efficacy on our primary and secondary outcomes for 155 participants who completed the baseline survey assessment and electronic pill bottle data (see Figure 1).

The observed participant characteristics for our final analytic sample are presented in Table 1. The analytic sample consisted of 31.6% (49/155) in the control group, 31.6% (49/155) in *Messages* and 36.8% (57/155) in the *Incentives* group. The combined analytic sample was predominately female 65.8% (102/155), married 56.1% (87/155) and employed at the time of the study 78.1% (121/155). The mean age of the combined sample was 33.9 years (SD 9.66), participants had an average of 2.67 children in their household (SD 2.55) and travel costs to the clinic represented 5.0% (SD 6.7%) of participants’ monthly disposable income on average.

**Table 1:** Participant characteristics across study groups at baseline

	(1)	(2)	(3)	(4)	(5)
	Control	Treatment Group 1	Treatment Group 2	Difference (1)−(2) (*p‐value*)	Difference (1)−(3) (*p‐value*)
Continuous variables: mean (standard deviation)					
Age	33.06	33.57	35.11	0.725	0.267
	(8.33)	(8.90)	(11.26)		
Health limitations	5.57	5.33	5.35	0.544	0.625
(*scale 1–6*)	(1.02)	(1.31)	(1.25)		
Number of children	2.37	2.94	2.70	0.376	0.909
	(2.13)	(3.27)	(2.16)		
Monthly income (USD)	59.20	78.02	72.95	0.546	0.812
	(119.29)	(81.71)	(116.55)		
Travel to clinic cost (USD)	3.79	3.52	3.33	0.961	0.552
	(3.72)	(3.49)	(2.39)		
Travel to clinic time (hours)	1.14	1.19	1.41	0.755	0.385
	(0.62)	(0.91)	(2.59)		
ART adherence motivation	38.33	37.73	38.58	0.189	0.305
(*scale 0–42*)	(3.01)	(3.26)	(3.31)		
Habit strength	17.45	17.88	17.32	0.238	0.396
(*scale 4–20*)	(1.99)	(2.52)	(2.73)		
Willingness to take risks	7.53	7.14	8.28	0.144	0.070
(*scale 0–10*)	(3.06)	(3.40)	(2.88)		
Willingness to delay rewards	8.37	8.39	8.21	0.839	0.737
(*scale 0–10*)	(3.24)	(2.60)	(3.07)		
Binary variables: percent (count)					
Male	34.69	36.73	31.58	0.650	0.601
	(17)	(18)	(18)		
Disclosed HIV status	87.76	81.63	82.46	0.606	0.715
	(43)	(40)	(47)		
Primary education or less	42.86	48.98	43.86	0.516	0.804
	(21)	(24)	(25)		
Married (or partnered)	63.27	46.94	57.89	0.117	0.735
	(31)	(23)	(33)		
Currently employed	71.43	81.63	80.70	0.465	0.545
	(35)	(40)	(46)		
Food insecure	22.45	26.53	21.05	0.508	0.625
	(11)	(13)	(12)		
**Observations**	**49**	**49**	**57**		

Intervention feasibility was measured using the rate of enrolment, randomization and study retention. Among eligible clients approached by the study team for participation, 86.6% (181/209) were initially enrolled in the study, 76.1% (159/209) were fully consented and randomized to one of the three study groups and 84.8% (135/159) of those randomized into a study group were retained in the study for the full 9‐month period including all three assessments and 9 months of adherence data collection. Across all three study groups, 92.6% (125/135) of participants reported being “very comfortable” using the electronic pill bottle cap for taking their ART medication at home. Among the two treatment groups, 92.4% (85/92) “strongly agreed” that the text messages provided important motivation and reminders for their daily ART adherence, and 96.0% (48/50) of *Incentives* participants reported strongly agreeing that the monthly prizes were easy to understand and provided motivation for taking their ART pills. Additionally, all participants either “strongly agreed” or “somewhat agreed” that the study benefits justified their time in the study, and all participants in the two treatment groups either “strongly” or “somewhat” agreed that the intervention helped them form an ART routine and that they will use this routine formation strategy for establishing other healthy routines in their life.

**Figure 2:**
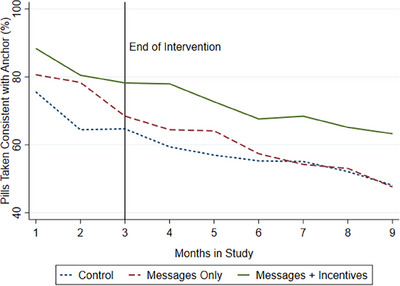
Cued pill‐taking by study group.

Figure 2 shows the study‐group level change in cued adherence during the 3‐month intervention and 6‐month post‐intervention period. The participant‐month measures were averaged over the 3‐month intervention, and during the intervention was higher in the *Messages* group (75.1% [SD 24.4%]) and *Incentives* group (81.0% [SD 20.2%]) than in the control group (68.5% [SD 28.9%]) (Table 2). In an OLS regression model that controls for all observable participant characteristics, cued adherence was higher in both *Messages* group (7.0% [95% CI –2.8 16.7; *p* = .06]) and *Incentives* group (12.2% [95% CI 2.2 22.2; *p* = .02]) during the intervention than in the control group (Table 2; column 2). After the intervention ended, cued adherence in the *Incentives* group remained significantly higher (14.2% [95% CI 1.1 27.2; *p* = .04]) than in the control group during months 4−6 (14.1% [95% CI −0.2 28.5; *p* = .05]) and months 7−9 (Table 2, columns 3−6).

**Table 2:** Effects on cued pill‐taking during and after the intervention period

	(1)	(2)	(3)	(4)	(5)	(6)
	Intervention period (Months 1–3)		Post‐intervention period (Months 4–6)		Post‐intervention period (Months 7–9)	
Average (SD) of cued pill‐taking:						
Control Group	68.5 (28.9)		57.5 (34.3)		51.5 (34.3)	
Treatment Group 1	75.1 (24.4)		61.3 (33.0)		53.0 (36.8)	
Treatment Group 2	81.0 (20.2)		71.7 (30.0)		66.4 (34.4)	
Regression model:						
Control Group	*Reference*		*Reference*		*Reference*	
Treatment Group 1	6.6	7.0	3.8	4.9	1.8	1.4
	[−4.1,17.3]	[−2.8,16.7]	[−10.1,17.8]	[−8.4,18.1]	[−13.5,17.0]	[−14.1,16.9]
Treatment Group 2	12.4^**^	12.2^**^	14.2^**^	14.2^**^	15.2^**^	14.1^*^
	[2.7,22.2]	[2.2,22.2]	[1.4,27.0]	[1.1,27.2]	[1.0,29.3]	[−0.2,28.5]
**Socio‐economic controls**		**X**		**X**		**X**
**Observations**	**155**	**155**	**145**	**145**	**135**	**135**

*Note*: This table presents ordinary least squares estimates and 95% confidence intervals [in brackets] for linear models of cued pill‐taking (defined as taking pills within +/− 1 hour from the typical time of day of participants’ chosen cue) during the 3‐month intervention and months 4−6 and 7−9 of the post‐intervention periods. Columns (1), (3) and (5) show the unadjusted effect of the two treatment groups relative to the control group, and columns (2), (4) and (6) show the effect of each treatment group after controlling for all observable socio‐economic characteristics summarized in Table 1.

^*^ *p* < 0.1; ^**^ *p* < .05.

**Figure 3:**
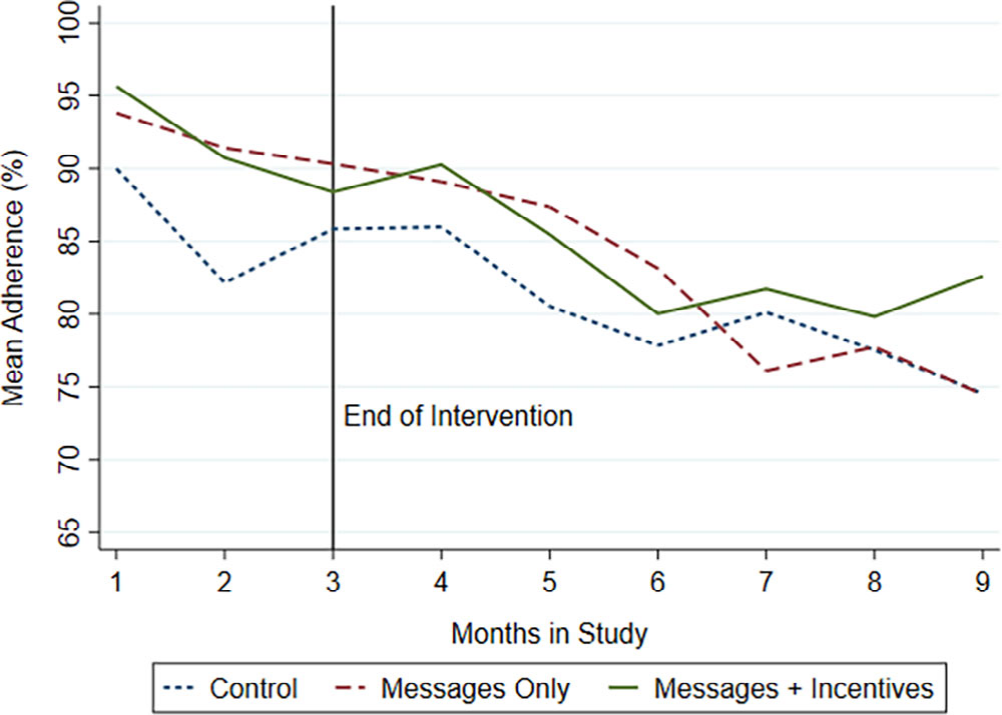
Mean ART adherence by study group.

Figure 3 presents the study‐group level changes in monthly mean adherence during the 3‐month intervention and 6‐month post‐intervention period. The participant‐month mean adherence measures were averaged over the 3‐month intervention, and Table 3 shows that the average adherence during the intervention was higher in the *Messages* group (91.3% [SD 14.5%]) and *Incentives* (91.7% [SD 11.2%]) relative to the Control group (86.0% [SD 18.7%]). An OLS regression model that controls for all observable participant characteristics (Table 3; column 2) shows higher mean adherence in both *Messages* group (5.7% [95% CI −0.2 11.5]; *p* = .05]) and *Incentives* group (5.4% [95% CI −0.3 11.0; *p* = .06]) during the intervention than the control group. During months 4−6, mean adherence remained significantly higher in the *Messages* group (6.9% [95% CI −1.2 15.1]; *p* = .05) than the control, and all groups had statistically equivalent mean adherence during months 7−9. Moderation analyses in Table 4 show that there was no observable treatment heterogeneity by whether someone took their medication in the morning or evening, nor by present bias or intrinsic motivation at baseline. The only difference in intervention effects was that cue changes during the intervention were associated with lower mean adherence during the intervention and post‐intervention periods.

**Table 3:** Effects on mean adherence during and after the intervention period

	(1)	(2)	(3)	(4)	(5)	(6)
	Intervention period (Months 1–3)		Post‐intervention period (Months 4–6)		Post‐intervention period (Months 7–9)	
Average (SD) of mean adherence:						
Control Group	86.0 (18.7)		81.7 (23.1)		78.9 (24.5)	
Treatment Group 1	91.3 (14.5)		86.8 (20.9)		77.0 (32.3)	
Treatment Group 2	91.7 (11.2)		86.1 (21.2)		82.7 (26.7)	
Regression model:						
Control Group	*Reference*		*Reference*		*Reference*	
Treatment Group 1	5.2	5.7^*^	5.1	6.9^*^	−1.9	1.6
	[−1.5,11.9]	[−0.2,11.5]	[−4.0,14.2]	[−1.2,15.1]	[−14.2,10.4]	[−11.7,15.0]
Treatment Group 2	5.7^*^	5.4^*^	4.5	3.7	3.9	3.4
	[−0.4,11.7]	[−0.3,11.0]	[−4.3,13.2]	[−5.1,12.4]	[−6.6,14.4]	[−7.5,14.2]
**Socio‐economic controls**		**X**		**X**		**X**
**Observations**	**155**	**155**	**145**	**145**	**135**	**135**

*Note*: This table presents ordinary least squares estimates and 95% confidence intervals [in brackets] for linear models of mean adherence during the 3‐month intervention and months 4−6 and 7−9 of the post‐intervention periods. Columns (1), (3) and (5) show the unadjusted effect of the two treatment groups relative to the control group, and columns (2), (4) and (6) show the effect of each treatment group after controlling for all observable socio‐economic characteristics summarized in Table 1.

^*^ *p* < 0.1.

**Table 4:** Heterogeneous treatment effects on mean adherence during the intervention

	(1)	(2)	(3)	(4)
	Intervention mean adherence (months 1−3)	Intervention mean adherence (months 1−3)	Intervention mean adherence (months 1−3)	Intervention mean adherence (months 1−3)
Treatment Group 1	4.3	5.8	3.3	4.2
	[−4.9,13.4]	[−1.9,13.4]	[−5.6,12.2]	[−7.6,16.0]
Treatment Group 2	6.2	7.3^**^	4.2	9.1^*^
	[−2.1,14.5]	[0.5,14.1]	[−2.7,11.1]	[−1.3,19.6]
Morning Cue	2.3			
	[−7.5,12.0]			
Treat 1 × Morning Cue	3.1			
	[−9.5,15.7]			
Treat 2 × Morning Cue	−1.4			
	[−13.0,10.3]			
Changed cue during intervention		5.5		
		[−2.3,13.2]		
Treat 1 × Changed Cue		−4.4		
		[−15.1,6.2]		
Treat 2 × Changed Cue		−10.1^*^		
		[−21.3,1.1]		
Present bias			−1.5	
			[−10.4,7.5]	
Treat 1 × Present Bias			3.1	
			[−9.4,15.7]	
Treat 2 × Present Bias			2.2	
			[−8.7,13.1]	
High Motivation at Baseline				9.4^*^
				[−0.8,19.6]
Treat 1 × High Motivation				1.6
				[−11.2,14.5]
Treat 2 × High Motivation				−7.5
				[−19.4,4.5]
**Observations**	**155**	**155**	**155**	**155**

*Note*: This table presents the ordinary least squares coefficient estimates and 95% confidence intervals [in brackets] for models of mean adherence during the 3‐month intervention. Each model includes identifiers for Treatment Group 1 and Treatment Group 2, as well as a moderating variable and the interaction between that variable and the treatment identifiers. A significant coefficient on any interaction term would indicate the presence of a moderating effect.

^*^ *p* < 0.1; ^**^ *p* < .05.

We did not observe an impact on the secondary outcomes of retention in care or viral load. Specifically, viral loads at baseline were already high in all three study groups (95.1% in the control group, 97.4% in *Messages* group and 97.8% in *Incentives*) and remained high throughout the study, with an average increase of 0.6 percentage points across all three groups. Retention in care was similarly high in all three groups, with only 4.5% (7/155) of participants no longer receiving HIV care from Mildmay at the end of the 9‐month study period.

## 4 DISCUSSION

In this study, we evaluate a novel approach to habit formation, and find promising preliminary improvements in habitual medication adherence among ART treatment initiators in a large HIV clinic in Uganda. Habits are a key strategy among those successfully managing chronic conditions and may hold the key to maintaining high medication adherence among ART treatment initiators. A common intervention strategy in the psychology literature called anchoring neglects many participants who drop out during the relatively long period it takes to form a habit, and during which many participants experience declining motivation. Complementing this strategy with insights from BE can allow even those who have low motivation to form healthy habits. Based on this synthesis approach detailed in Stecher and Linnemayr (2021), we use SMS reminders to keep the salience of habit formation high, and in a second intervention group additionally provide rewards to counter present bias. Importantly, we use the novel strategy of conditioning the incentives on cued adherence (i.e. adherence in line with a person's anchoring plan rather than mean adherence as in most existing incentive‐based interventions) to directly incentivize habit formation. SMS reminders and incentives are only used during the relatively short intervention period, with the goal to establish pill‐taking habits that will be maintained after the intervention ends.

Our intervention successfully increased habit formation, as evidenced by increased cued ART adherence in both intervention groups compared to the control. However, cued adherence was only maintained for those in the incentives group, indicating that incentives may be needed for habit formation. Increased mean adherence during the intervention in both intervention groups dissipates after several months, possibly because people pay more attention to their (timely) adherence when the intervention is new to them. A longer post‐intervention observation period could potentially reduce any such novelty effect, and allow for frequently requested cue changes, which in Supplementary Analyses we find to reduce the intervention effect (see Supplementary Tables). We observe less than 5% attrition, compared to habit formation interventions in the existing literature that often see 40% or more participant drop out. This high acceptance of the study is also supported by overwhelmingly positive perceptions of study participants.

Three changes in the study design may increase the impact of our habit formation approach in a future, fully powered RCT: first, given the high adherence rates among participants in the current study, the intervention should be targeted to those in need of adherence support, such as those failing to show drug refills, possibly by first measuring adherence and then restricting the intervention to those showing low adherence. Second, a significant fraction (16.1%; 25/155) of study participants changed their anchor and/or the anchor time during the intervention. This resulted in a shortened intervention period using the new anchor, and may have contributed to a smaller intervention effect in this pilot study. In future studies, the interventionist should make sure that participants understand correctly how to pick an anchor that works for them, and anchor changes should be both expected and integrated into the study design. A fully powered RCT should also include a longer post‐intervention observation period to evaluate how well habits stick, and find out when/why habits are interrupted, whether people manage to re‐establish them on their own and how to get them back into their previously established habit. Lastly, there may be room for increasing the efficacy of the individual intervention components such as through more personalized or otherwise tailored messages, or more frequent or larger incentives over a longer intervention period. Alternatively, an adaptive design could be used that first offers the relatively low‐cost messages to participants, and only provides the more resource‐intensive incentives to those not benefitting from the messages alone (albeit in our study the incentives only cost about $3.60 USD per participant, which is likely cost‐effective but should be investigated through a formal cost‐effectiveness analysis as part of a fully powered study).

Strengths of our study include a rigorous conceptual foundation based on the framework developed in Stecher and Linnemayr (2021), a robust statistical analysis based on an RCT design, and our measure of cued medication adherence to assess whether a person is taking their pills in accordance with their anchoring plan. Cued adherence is an objective measure of habit strength [34], and is more strongly associated with long‐term adherence and improved health outcomes than the traditional measure of mean adherence [11, 35]. Limitations consist of our statistical analysis being based on a small pilot study that was not powered for all outcomes. Also, the intervention took place in a single HIV clinic in Uganda, but we believe the intervention site (and hence our results) to be fairly representative of many urban clinics in sub‐Saharan Africa.

## 5 CONCLUSIONS

The approach of combining incentives based on behavioural insights to supplement the existing anchoring approach to habit formation was found feasible and acceptable in our pilot study among ART treatment initiators, and showed promising preliminary results. It now needs to be evaluated in a fully powered study to further our understanding of the habit formation process, which will yield important insights not only for HIV medication adherence but also for a range of other conditions requiring chronic disease self‐management. Any such future intervention at scale should include a cost‐effectiveness analysis, as we hypothesize that the approach of a short‐term intervention to bring about long‐lasting changes in medication adherence is likely to be cost‐effective.

## COMPETING INTERESTS

The authors declare no competing interests.

## AUTHORS’ CONTRIBUTIONS

SL and CS co‐led the analyses and co‐wrote the article; IG oversaw data collection and management across the survey rounds and contributed to the analysis. SL secured the funding and designed the overall study. All authors reviewed and provided input to the final draft. SL had final responsibility for the decision to submit for publication. SL and CS both directly accessed and verified the underlying data reported in the manuscript.

## FUNDING

This pilot RCT was funded by the National Institutes of Mental Health in the United States (R34MH122331, PI Linnemayr).

## Supporting information


**Table S1**: Heterogeneous Treatment Effects on Cued Pill Taking During the Intervention
**Table S2**: Heterogeneous Treatment Effects on Mean Adherence During the Intervention

## Data Availability

Data will be shared with the research community when papers using the data have been accepted for publication or at the end of the award period (including the first no‐cost extension period), whichever occurs sooner.
