# Supplementary material for: INcentives and ReMINDers to Improve Long‐Term Medication Adherence (INMIND): impact of a pilot randomized controlled trial in a large HIV clinic in Uganda
Source: J Int AIDS Soc. 2024 Jun 25;27(6):e26306. doi: 10.1002/jia2.26306 (PMC11197960; doi:10.1002/jia2.26306)
Supplement: Supplementary file 1 — Table S1: Heterogeneous Treatment Effects on Cued Pill Taking During the Intervention Table S2: Heterogeneous Treatment Effects on Mean Adherence During the Intervention [file JIA2-27-e26306-s001.docx]

**Table S1: Heterogeneous Treatment Effects on Cued Pill Taking During the Intervention**

|  | (1) | (2) | (3) | (4) |
| --- | --- | --- | --- | --- |
|  | Intervention Cued  Pill Taking | Intervention Cued  Pill Taking | Intervention Cued  Pill Taking | Intervention Cued  Pill Taking |
| Treatment Group 1 | 0.026 | -0.001 | 0.087 | 0.007 |
|  | [-0.15,0.18] | [-0.19,0.19] | [-0.11,0.21] | [-0.15,0.16] |
| Treatment Group 2 | 0.217 | 0.313^**^ | 0.340^**^ | 0.246^**^ |
|  | [-0.05,0.27] | [0.01,0.31] | [0.04,0.31] | [0.01,0.24] |
| Morning Cue | 0.035 |  |  |  |
|  | [-0.16,0.20] |  |  |  |
| Treat 1 x Morning Cue | 0.157 |  |  |  |
|  | [-0.12,0.33] |  |  |  |
| Treat 2 x Morning Cue | 0.062 |  |  |  |
|  | [-0.18,0.25] |  |  |  |
| Changed Cue During Intervention |  | 0.084 |  |  |
|  |  | [-0.14,0.22] |  |  |
| Treat 1 x Changed Cue |  | 0.154 |  |  |
|  |  | [-0.15,0.34] |  |  |
| Treat 2 x Changed Cue |  | -0.090 |  |  |
|  |  | [-0.27,0.16] |  |  |
| Present Bias |  |  | 0.130 |  |
|  |  |  | [-0.11,0.24] |  |
| Treat 1 x Present Bias |  |  | 0.067 |  |
|  |  |  | [-0.18,0.27] |  |
| Treat 2 x Present Bias |  |  | -0.134 |  |
|  |  |  | [-0.29,0.13] |  |
| High Motivation at Baseline |  |  |  | -0.096 |
|  |  |  |  | [-0.20,0.10] |
| Treat 1 x High Motivation |  |  |  | 0.163 |
|  |  |  |  | [-0.11,0.32] |
| Treat 2 x High Motivation |  |  |  | 0.016 |
|  |  |  |  | [-0.18,0.20] |
| **Observations** | **155** | **155** | **155** | **155** |

Note: This table presents the ordinary least squares coefficient estimates and 95% confidence intervals [in brackets] for models of cued pill-taking (defined as taking pills within +/- 1 hour from the typical time of day of participants’ chosen cue) during the 3-month intervention. Each model includes identifiers for Treatment Group 1 and Treatment Group 2, as well as a moderating variable and the interaction between that variable and the treatment identifiers. A significant coefficient on any interaction term would indicate the presence of a moderating effect. ^**^ *p* < 0.05

**Table S2: Heterogeneous Treatment Effects on Mean Adherence During the Intervention**

|  | (1) | (2) | (3) | (4) |
| --- | --- | --- | --- | --- |
|  | Intervention Mean Adherence | Intervention Mean Adherence | Intervention Mean Adherence | Intervention Mean Adherence |
| Treatment Group 1 | 0.043 | 0.058 | 0.033 | 0.042 |
|  | [-0.05,0.13] | [-0.02,0.13] | [-0.06,0.12] | [-0.08,0.16] |
| Treatment Group 2 | 0.062 | 0.073^**^ | 0.042 | 0.091^*^ |
|  | [-0.02,0.14] | [0.01,0.14] | [-0.03,0.11] | [-0.01,0.20] |
| Morning Cue | 0.023 |  |  |  |
|  | [-0.07,0.12] |  |  |  |
| Treat 1 x Morning Cue | 0.031 |  |  |  |
|  | [-0.09,0.16] |  |  |  |
| Treat 2 x Morning Cue | -0.014 |  |  |  |
|  | [-0.13,0.10] |  |  |  |
| Changed Cue During Intervention |  | 0.055 |  |  |
|  |  | [-0.02,0.13] |  |  |
| Treat 1 x Changed Cue |  | -0.044 |  |  |
|  |  | [-0.15,0.06] |  |  |
| Treat 2 x Changed Cue |  | -0.101^*^ |  |  |
|  |  | [-0.21,0.01] |  |  |
| Present Bias |  |  | -0.015 |  |
|  |  |  | [-0.10,0.07] |  |
| Treat 1 x Present Bias |  |  | 0.031 |  |
|  |  |  | [-0.09,0.16] |  |
| Treat 2 x Present Bias |  |  | 0.022 |  |
|  |  |  | [-0.09,0.13] |  |
| High Motivation at Baseline |  |  |  | 0.094^*^ |
|  |  |  |  | [-0.01,0.20] |
| Treat 1 x High Motivation |  |  |  | 0.016 |
|  |  |  |  | [-0.11,0.15] |
| Treat 2 x High Motivation |  |  |  | -0.075 |
|  |  |  |  | [-0.19,0.05] |
| **Observations** | **155** | **155** | **155** | **155** |

Note: This table presents the ordinary least squares coefficient estimates and 95% confidence intervals [in brackets] for models of mean adherence during the 3-month intervention. Each model includes identifiers for Treatment Group 1 and Treatment Group 2, as well as a moderating variable and the interaction between that variable and the treatment identifiers. A significant coefficient on any interaction term would indicate the presence of a moderating effect. ^*^ *p* < 0.1; ^**^ *p* < 0.05
